# A Review on the Reactivity of 1-Amino-2-Nitroguanidine (ANQ)

**DOI:** 10.3390/molecules24193616

**Published:** 2019-10-08

**Authors:** Jinghua Wang, Meng Cai, Fengqi Zhao, Kangzhen Xu

**Affiliations:** 1 School of Chemical Engineering, Northwest University, Xi’an 710069, China; 201831731@stumail.nwu.edu.cn (J.W.); cm201820747@163.com (M.C.); 2 Xi’an Modern Chemistry Research Institute, Xi’an 710065, China; zhaofqi@163.com

**Keywords:** 1-Amino-2-nitroguanidine (ANQ), energetic materials, derivatives, energetic properties

## Abstract

1-Amino-2-nitroguanidine (ANQ) is a high-energy nitrogen-rich compound with good detonation properties and low sensitivities. ANQ has only a central carbon atom with three small groups around it, including an amino, a hydrazine and a nitroxyl group. Though the molecular structure of ANQ is very simple, its reactivity is surprisingly abundant. ANQ can undergo various reactions, including reduction reaction, acylation reaction, salification reaction, coordination reaction, aldimine condensation reaction, cyclization reaction and azide reaction. Many new energetic compounds were purposely obtained through these reactions. These reactions were systematically summarized in this review, and detonation properties of some energetic compounds were compared. In the field of energetic materials, ANQ and some derivatives exhibit good application prospects.

## 1. Introduction

**Figure 1:**
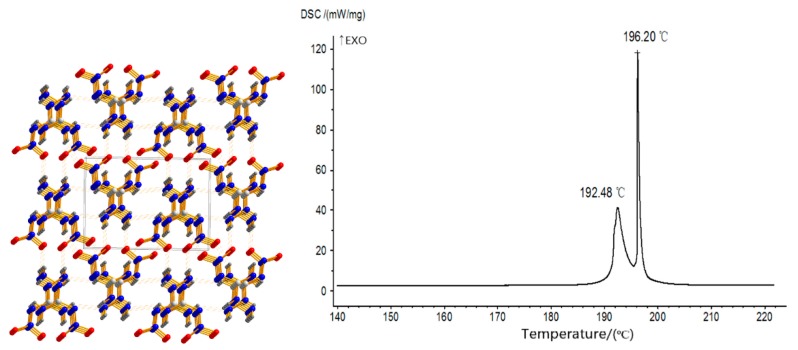
Packing diagram and DSC curve of 1-Amino-2-nitroguanidine.

1-Amino-2-nitroguanidine (ANQ, **1**) was first synthesized by Phillips in 1928 [1] and has played an important role in the synthesis of valuable biologically active compounds, such as pesticides and medicines [2,3,4,5,6,7,8,9,10,11,12,13,14,15,16]. In recent years, Klapötke has done a lot of research on the application of ANQ in high-energy nitrogen-rich materials, which began to attract significant interest in the field of energetic materials [17,18]. The molecular structure of ANQ is very simple, containing only an amino, a hydrazino and a nitro group around the central carbon atom and its non-hydrogen atoms are almost on the same plane. Moreover, the bond angles of three main groups in the molecule are about 120° with a relatively uniform distribution, indicating that the structure is comparatively regular and conforms to the structural requirements of the energetic materials. One kind of intramolecular and five kinds of intermolecular hydrogen bonds exist in ANQ, which extend it into a two-dimensional planar structure. Meanwhile, a stable spatial structure of ANQ is formed by electrostatic attraction and van der Waals force between layers, as seen in Figure 1. The crystals of ANQ are transparent yellow and crystallize in the monoclinic crystal system with the space group *P*2_1_/*c* [19]. ANQ can dissolve in water to the extent of 0.34 % (mass fraction) at 20 °C and 3.0% at 70 °C, and is easily soluble in sodium hydroxide solution, but cannot be readily dissolved in common organic solvents [1]. The decomposition behavior of ANQ presents two intense exothermal processes, and peak temperatures at a heating rate of 5 °C min^−1^ are 192.48 and 196.20 °C, respectively, as presented in Figure 1 [20]. Furthermore, the self-accelerating decomposition temperature (*T*_SADT_) and thermal explosion critical temperature (*T*_b_) for ANQ are 184.5 and 192.7 °C [20]. ANQ has good features, such as high nitrogen content (58.8%), big density (1.767 g cm^−3^), decent detonation velocity (8.25 km s^−1^) and detonation pressure (30.7 GPa) [21,22], suggesting that ANQ is a potential high-energy material.

**Figure 2:**
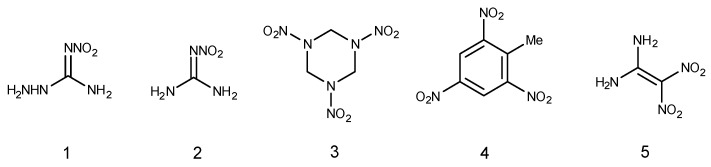
Structures of ANQ, NQ, RDX, TNT, and FOX-7.

The detonation performances of ANQ and other common energetic materials for comparison are shown in Table 1, including nitroguanidine (NQ, **2**), 1,3,5-trinitroperhydro-1,3,5-triazine (RDX, **3**), 2,4,6-trinitrotoluene (TNT, **4**), and 1,1-diamino-2,2-dinitroethylene (FOX-7, **5**) (Figure 2) [18,21,22,23,24,25,26,27,28,29,30,31,32,33,34]. ANQ, as a suitable replacement for the high-energy explosive RDX, has similar detonation performances to NQ, RDX and FOX-7 and superior detonation performance to TNT.

**Scheme 1:**
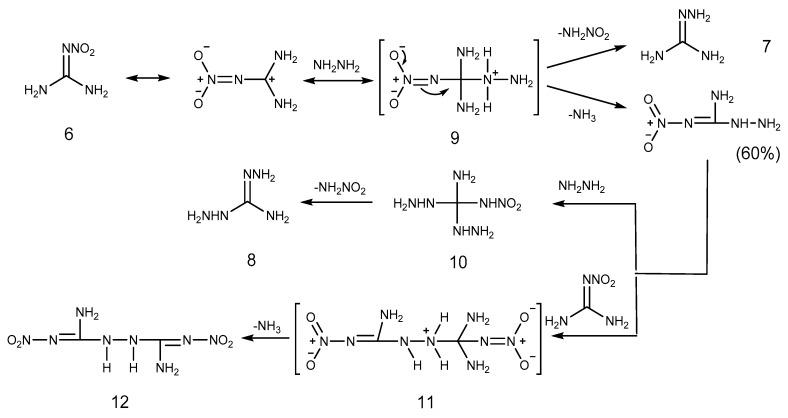
Synthetic route of ANQ from nitroguanidine.

**Scheme 2:**
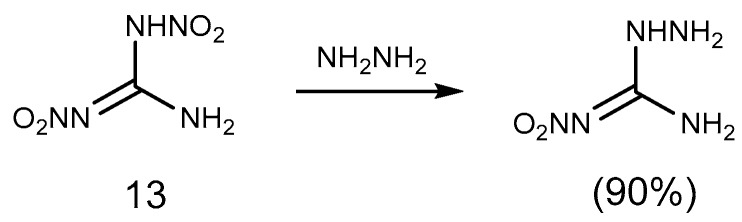
Synthetic route of ANQ from 1,2-dinitroguanidine.

**Scheme 3:**
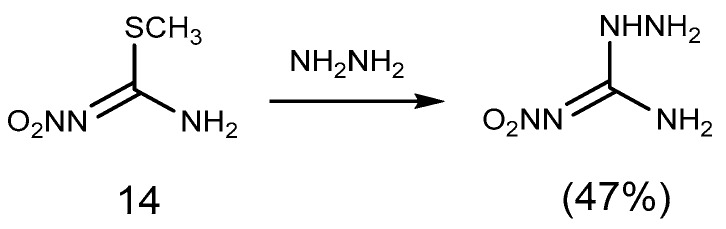
Synthetic route of ANQ from 2-methylnitroisothiourea.

To the synthesis of ANQ, Phillips first reported ANQ with the yield of 50% through the reaction of hydrazine sulfate and nitroguanidine (**6**) in aqueous ammonia at 50–60 °C [1]. Castillo-Meléndez improved the method by dissolving nitroguanidine and 80% hydrazine hydrate in water at 55 °C for 15 min with the yield of 60%, but the reaction temperature and reaction time must be precisely controlled [35]. Two side-products, including aminoguanidine (**7**) and diaminoguanidine (**8**), would be found in the reaction [18]. Assuming an addition-elimination mechanism, a probable reason is that there are two competitive reactions in the deamination condensation process of intermediate **9**, which containing a tetrasubstituted carbon atom is formed by the reaction of nitroguanidine and hydrazine hydrate. One is that the intermediate **9** loses ammonia to form ANQ; the other is that the intermediate **9** loses nitramine to form aminoguanidine (**7**). In addition, using a larger excess of hydrazine hydrate, the resulting ANQ can continue to react with hydrazine hydrate forming intermediate **10**, which eliminates nitramine to obtain 1,2-diaminoguanidine (**8**) [18,36]. Using a larger excess of nitroguanidine, ANQ continues to react with nitroguanidine to form intermediate **11**, and hydrazobis (nitroformamidine) (**12**) is further obtained, as seen in Scheme 1 [37]. Another method was reported by Astrat’yev in 2002 [38]. In this report, ANQ was readily prepared using 1,2-dinitroguanidine (**13**) and 25% hydrazine hydrate in aqueous at 70–80 °C for 2 h with the yield of 90% (Scheme 2), but compound **13** is more difficult to obtain, so the method is rarely used for the synthesis of ANQ. In 2016, Jia also reported a new synthesis method for ANQ based on the reaction of 2-methyl-3-nitroisothiourea (**14**) with hydrazine hydrate (80%) at 40–70 °C (Scheme 3), which possessed short synthesis steps and simple post-processes, but the yield was low, only 47% [39,40].

**Scheme 4:**
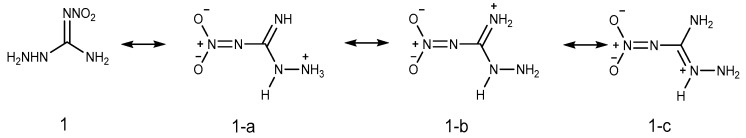
Resonance hybrid of ANQ.

**Figure 3:**
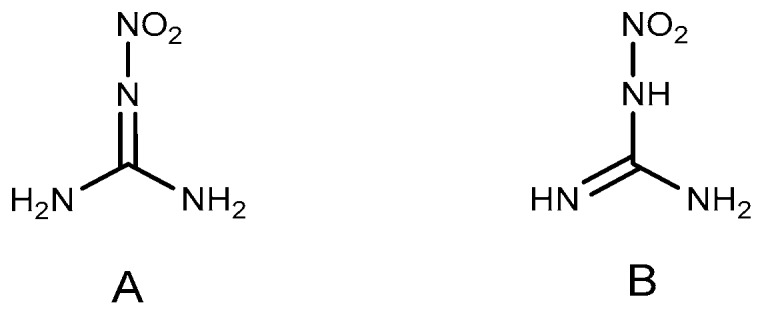
Correct nitrimine structure (**A**) and wrong nitramine structure (**B**) of nitroguanidine.

The molecular structure of ANQ can be regarded as a resonance hybrid between the mesomer 1 and 1-a, 1-b, 1-c, as seen in Scheme 4 [41]. It is noted that the structure is similar to that of NQ. We know that NQ has long been considered to exist in two isomers. However, in terms of reactivity and spectroscopic data, the structure of NQ is not a nitramine (**B**), but a nitrimine (**A**), as seen in Figure 3. This fact suggests that ANQ is a nitrimine structure as well [23]. Due to the misunderstanding of the structure of ANQ, it has long been considered as an amphoteric substance [19,42,43]. However, according to the nitrimine structure of ANQ, it is an alkaline substance and is easily protonated. Of course, owing to its electron-withdrawing nitro group and electron-donating hydrazino group, ANQ possesses a high reactivity, and the reactivity can be described as the following four aspects: (a) due to the protonation of the terminal amino group, ANQ can generate salification reaction; (b) due to the high electron density of amino and nitro groups, it can be used as a good ligand in many coordination reactions; (c) the nucleophilic substitution of the hydrazino group; (d) other reactions of the nitro group and the amino group.

## 2. Reactivity of ANQ

### 2.1. Reduction Reaction

**Scheme 5:**
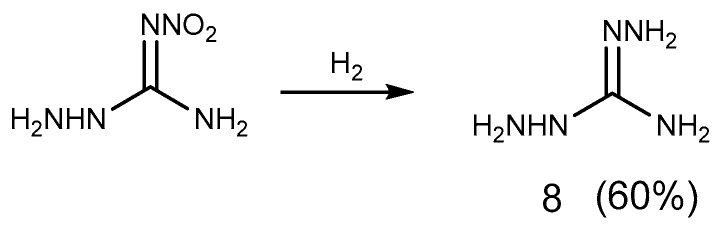
Reduction reaction of ANQ.

The reduction of ANQ by hydrogen can obtain diaminoguanidine (**8**) [1], as seen in Scheme 5, but no relevant papers confirmed the feasibility of the synthesis method. However, compound **8** can be obtained in the synthesis process of ANQ, as seen in Scheme 1, if the reaction temperature and time are not properly controlled [18].

### 2.2. Acylation Reaction

**Scheme 6:**
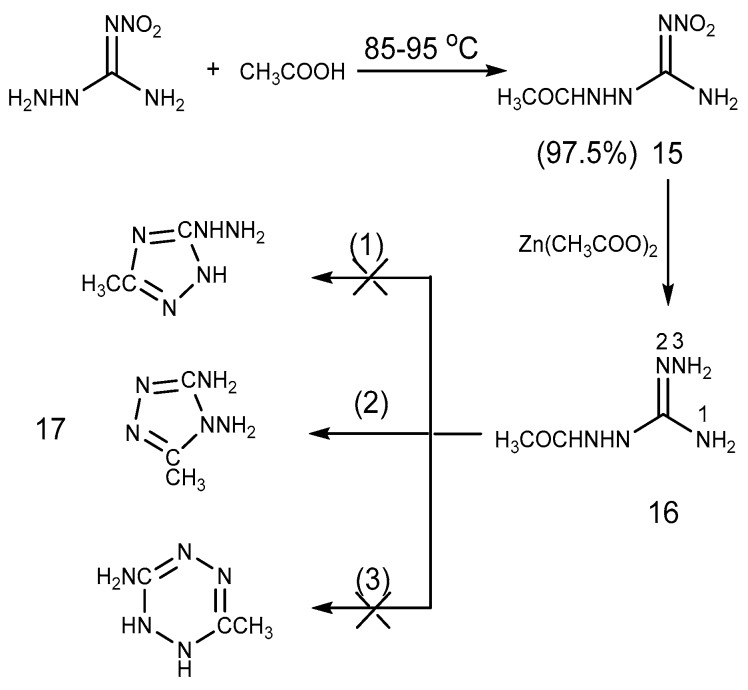
Acylation of ANQ with acetic acid.

**Scheme 7:**
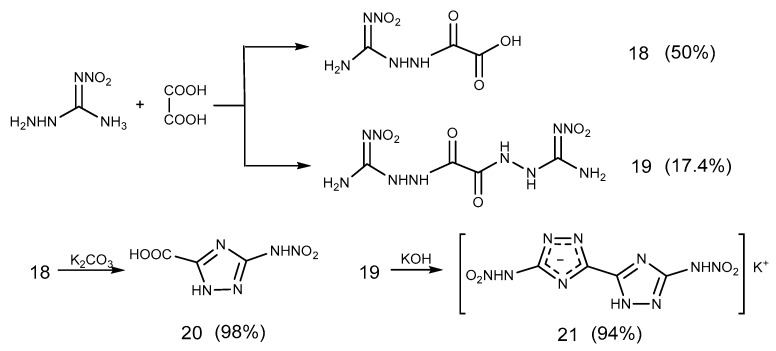
Acylation of ANQ with oxalic acid.

**Scheme 8:**
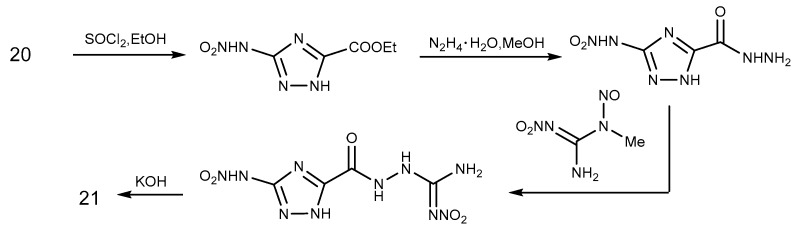
Reaction of compound 21 from compound 20.

In the temperature range of 85 to 90 °C, ANQ could be acylated by acetic anhydride-acetic acid to form 1-acetamido-2-nitroguanidine (**15**) with a very high yield (97.5%) [44]. However, at room temperature, ANQ could react with acetic acid to form compound **15** which was easier to be hydrogenated than ANQ, and 1-acetamido-2-aminoguanidine (**16**) also could be obtained in the presence of zinc acetate. Theoretically, the cyclization reaction of compound **16** may occur at N^1^, N^2^ or N^3^ and lead to the formation of the corresponding three products. However, the evidence suggested that this ring-closure reaction occurred mainly at N^2^, and 3-methyl-4,5-diamino-l, 2,4-triazole (**17)** was easily obtained, as seen in Scheme 6 [45]. Reaction with oxalic acid in aqueous solution at 92–94 °C for 8 h resulted in the corresponding mono- and di-hydrazides, compounds **18** and **19**. Both of them were able to be cyclized to form nitroaminotriazole derivatives (**20**, **21**) in the presence of alkaline material, as seen in Scheme 7 [46]. 5,5′-Bi(3-nitroamino-1,2,4-triazole) potassium salt (**21**) can also be obtained from 5(3)-nitroamino-1,2,4-triazole-3(5)-carboxylic acid (**20**) after a series of reactions, as seen in Scheme 8 [46].

### 2.3. Salification Reaction

**Scheme 9:**
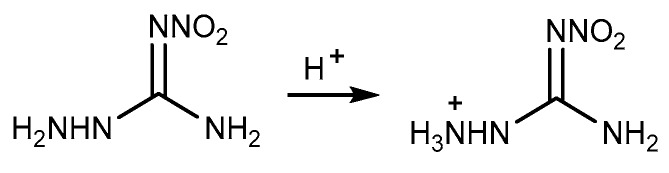
ANQ and its protonated cation.

Since the hydrazine group is electron-donating, ANQ is easily protonated to form cations under strong acidic conditions, as seen in Scheme 9, which leads to the formation of the inorganic salts (**22**–**27**) and organic salts (**28**–**42**) of ANQ. For example, ANQ can react with nitric acid to form 1-amino-2-nitroguanidinium nitrate (ANGN, **22**), which crystallizes in the triclinic crystal system with space group *P*ī [47]. In addition, the halides as well as the sulfate salt also can be prepared by dissolving ANQ in dilute aqueous solutions of the respective mineral acids HCl, HBr, HI and H_2_SO_4_. However, it cannot react with HF, since the acidic strength of HF in aqueous solution is the weakest among all the hydrogenides, and HF is not able to protonate ANQ in aqueous solution [48]. Especially, the detonation pressure (42.7 GPa) and detonation velocity (9.551 km s^−1^) of ANGN are both superior to those of RDX, which can be considered as a potential candidate for high-energy-density compounds [18].

The hydrochloride salt of ANQ can react with organic silver salts to obtain some organic salts of ANQ, such as 2,4,5-trinitroimidazole salt (**28**), 5-nitro-1,2,3,4-tetrazole salt (**29**) [49], 4-nitroamino-1,2,4-triazole salt (**30**) [50], 3,5-dinitro-1,2,4-triazole salt (**31**) [51], 3-hydroxy-5-nitrotetrazole salt (**32**) [52] and dinitramide monohydrate salt (**33**) [18]. Those organic salts contain a large amount of C-N and N-N bonds, so the formation enthalpies and detonation characteristics of these materials are greatly improved. For the above compounds, except **33**, thermal decomposition processes of these compounds present an evident sharp exothermic peak. The peak temperatures fall in the range 108–271.1 °C, and the heat releases are over the range of 1167–2589 J g^−1^. Compounds **29** and **32** have lower melting points as 83.2 and 93.8 °C, and compound **32** has the highest critical explosion temperature at 162.9 °C. The densities, standard molar formation enthalpies, detonation pressures and detonation velocities of these compounds (**28**–**33**) distribute in 1.59–1.85 g cm^−3^, 264.7–551.4 kJ mol^−1^, 26.6–37.7 GPa and 8.05–9.17 km s^−1^, respectively [53].

**Scheme 10:**
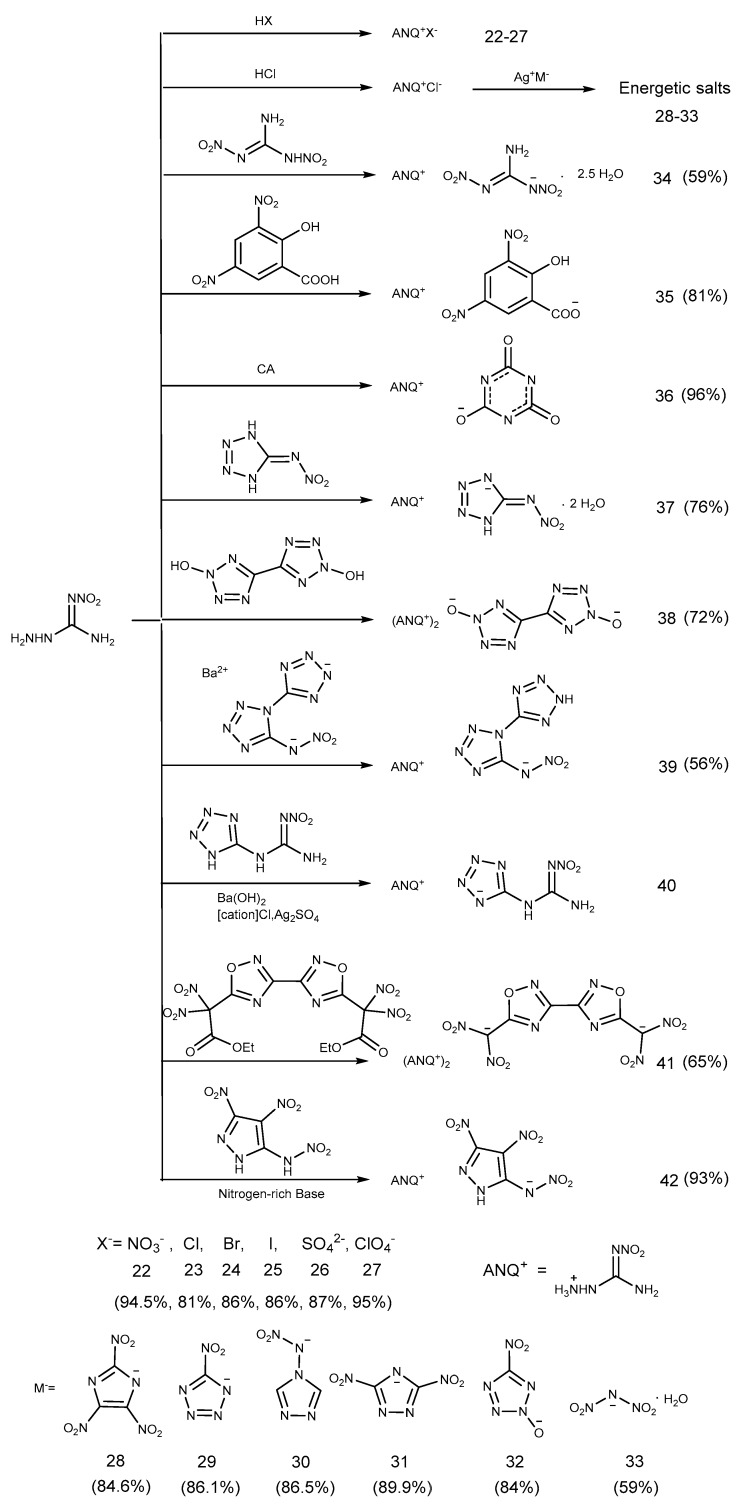
Salification reaction of ANQ.

ANQ reacted with dinitroguanidine to obtain corresponding dinitroguanidinium salt (**34**) [18]. Li found that 1-amino-2-nitroguanidinium 3,5-dinitrosalicylate (ANQ·DNS) (**35**) can be obtained by using 3,5-dinitrosalicylic acid in aqueous solution at 80 °C [19]. Analogously, the treatment of ANQ with azine compound cyanuric acid (CA) resulted in ANGDTO (**36**) (*ρ* = 1.75 g cm^−3^) [54]. Moreover, ANQ can react with tetrazole to form corresponding 5-nitriminotetrazolium salt (**37**) [18], 5,5′-bis(tetrazole-2-oxide) salt (**38**) [55], 1-(2*H*-tetrazol-5-yl)-5-nitrosyltetrazolium salt (**39**) [56] and 5-nitroguanidyltetrazolium salt (**40**) [57]. Compound **39** shows an ultrahigh detonation velocity (9.432 km s^−1^), indicating that it can be used as a high explosive. In addition, the reaction of ANQ with 3,3′-bis(1,2,4-oxadiazolyl)-5,5′-bis(2,2′-dinitro)-diacetic acid diethyl ester formed aminonitroguanidinium nitrogen-rich salts (HANG_2_-DBO, **41**) in aqueous methanol solution [58]. Energetic salt **42** can be synthesized from the reaction of 5-nitramino-3,4-dinitropyrazole with ANQ. The result indicates that compound **42** exhibits good performances with high detonation pressure and detonation velocity as 39.5 GPa and 9.237 km s^−1^ [59]. All reactions are gathered in Scheme 10.

### 2.4. Coordination Reaction

**Scheme 11:**
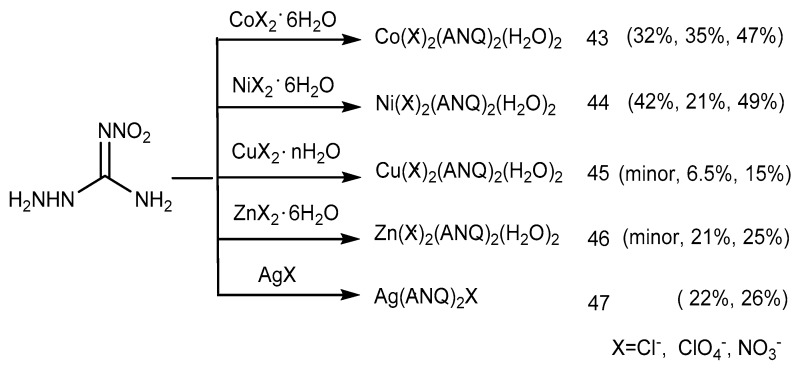
Coordination reaction of ANQ.

**Scheme 12:**
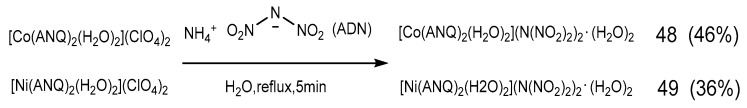
Complicated coordination reaction of ANQ.

In 1928, Phillips firstly reported that ANQ reacted with nickel sulfate to form a nickel complex, which owned a high decomposition temperature of 220 °C [1]. ANQ can form energetic metal complexes [M^2+^(ANQ)_2_(X^−^)_2_(H_2_O)_n_ (n = 0, 2), in the case of M = Co, Ni, Cu, Zn (**43**–**46**), and M^+^(ANQ)_2_(X^−^)(H_2_O)_y_ in the case of M = Ag (**47**)] with perchlorate or nitrate solution of transition metal [60]. Herein, nitrate, perchlorate and chloride are the anions of the complex, and ANQ is the ligand for the synthesis of high energy transition metal complexes, as seen in Scheme 11. Complexes M^2+^(ANQ)_2_(X^−^)_2_(H_2_O)_4_ (**48**, **49**) with X=N(NO_2_)_2_ can be synthesized in the case of M=Co and Ni, in a stoichiometric ratio of perchlorate complexes of cobalt (nickel): Ammonium dinitramide (ADN) = 1:2, as seen in Scheme 12 [60]. All those complexes containing perchlorate, nitrate and chloride crystallize as dihydrates, except silver complexes (**47**) are water free. Dinitramide crystallizes as tetrahydrate (**48**, **49**), and most of the complexes containing crystal water can be dehydrated without decomposition. In these complexes, the nickel complex is the most stable, having the highest decomposition temperature of 250 °C, while the copper and silver complexes decompose at a low temperature of about 77 °C. Compared with nitrates and ammonium dinitramide (ADN), the decomposition temperatures of chlorides and perchlorates tend to increase. Moreover, all the complexes present high impact and friction sensitivities, especially the solvate water free silver complex (**47**) and the copper nitrate complex [60]. ANQ can also have coordination reaction with cobalt (II), nickel (II), copper (II) and 2-hydroxybenzaldehyde or 4-(dimethylamino)benzaldehyde [61].

### 2.5. Aldimine Condensation Reaction

**Scheme 13:**
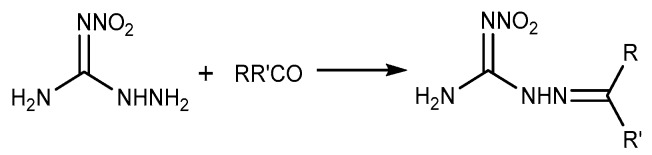
Aldehyde ketone reaction of ANQ.

**Scheme 14:**
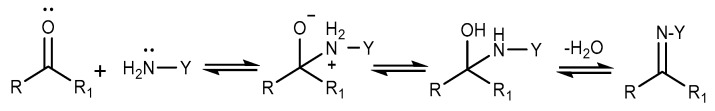
Reaction mechanism of ANQ with aldehydes and ketones under acidic conditions.

**Scheme 15:**
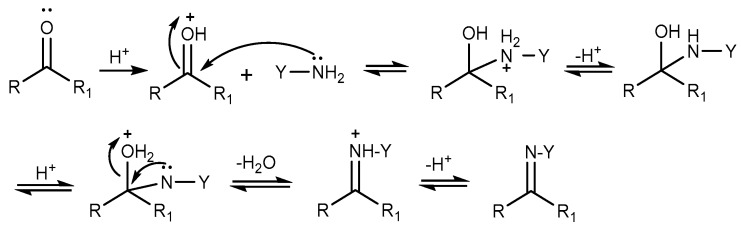
Reaction mechanism of ANQ with aldehydes and ketones under alkaline conditions.

ANQ can undergo condensation reactions with aldehydes and ketones to form hydrazine or Schiff base compounds. Phillips found that ANQ can react with aldehydes and ketones by using an acid or a base as catalyst, as shown in Scheme 13 [1]. When an acid is used as catalyst, its hydrogen ion can be combined with a carbonyl group to form an oxonium salt, thereby increasing the electrophilicity of the carbonyl group [62]. The nucleophilic addition-elimination reaction mechanisms under acid-base conditions are shown in Scheme 14 and Scheme 15.

**Scheme 16:**
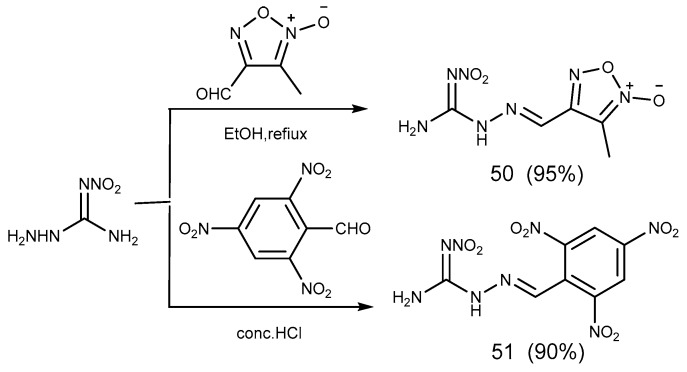
Aldehyde ketone reaction of ANQ.

ANQ reacted with 3-methyl-4-furoxancarbaldehyde and 2,4,6-trinitrobenzaldehyde leading to the formation of 3-methyl-4-((2-(*N*′-nitrocarbamimidoyl)hydrazono)methyl)-1,2,5-oxadiazole-2- oxide (**50**) and *N*′-nitro-2-(2,4,6-trinitrobenzylidene)hydrazinecarboximidamide (**51**), as seen in Scheme 16 [63]. Only hydrazino group of ANQ could react with aldehyde group, and no condensation reaction of amino group with aldehyde group was found. This may be because the strong electron-withdrawing effect of nitro group reduced the reactivity of amino group.

**Scheme 17:**
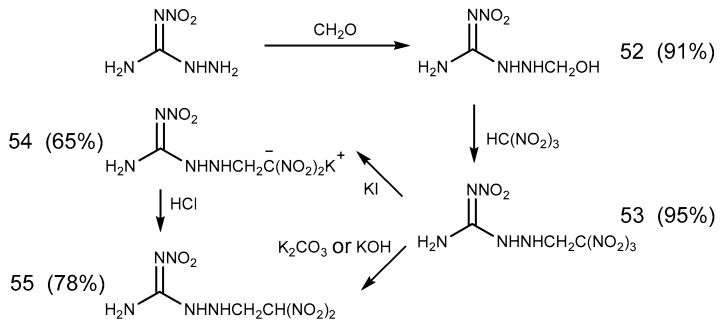
Synthesis of 1-hydroxymethylamino-2-nitroguanidine and its derivatives.

Additionally, the treatment of ANQ with formaldehyde resulted in the formation of 1-hydroxymethylamino-2-nitroguanidine (**52**). The terminal hydroxyl function of compound had high activity and could further react with nitroform in methanol, ethanol or water to obtain 2-nitro-1-(2,2,2-trinitroethylamino)guanidine (TNEANG) (**53**) [64,65]. Compound **53** was a typical trinitromethyl derivative, which could be reduced by iodide to form a potassium salt of 1-(2,2-dinitroethylamino)-2-nitroguanidine (**54**) [65]. It was also able to react with denitration agents such as K_2_CO_3_ or KOH, followed by acidification with hydrochloric acid to obtain 1-(2,2-dinitroethylamino)-2-nitroguanidine (DNEANG) (**55**) containing a dinitromethyl structure. The separation phase of potassium salt **54** was successfully eliminated, increasing the yield of **55** from 44% to 78%, as seen in Scheme 17 [53,64,65,66,67]. The detonation properties of TNEANG (**53**) and DNEANG (**55**) have not been reported, but their melting points are as low as 95–96 °C and 93–94 °C respectively, which are promising as melting phase for melt casting explosives [64].

**Scheme 18:**
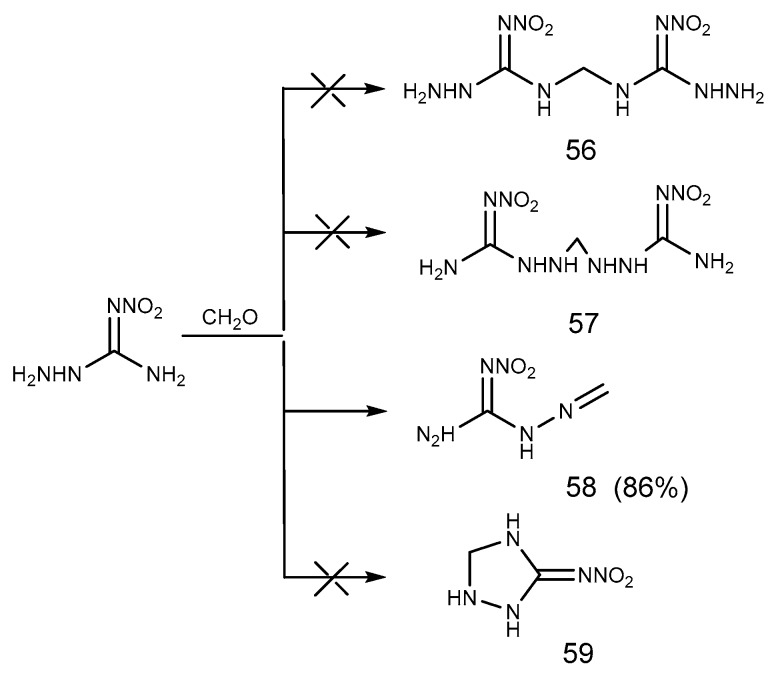
Reaction of ANQ with formaldehyde.

Based on the reaction of nitroguanidine and formaldehyde to form methylene dinitroguanidine, our research group hoped that designing a route of linking two molecules of ANQ with methylene to synthesize a symmetric energetic compound **56** or **57**, but failed even though various methods were attempted. However, methyleneaminonitroguanidine (MANG) (**58**), as shown in Scheme 18, was unexpectedly synthesized with a high yield of 86% [68]. The reason should be that the amino of the hydrazino group is more nucleophilic. The nucleophilic addition reaction firstly occurs since the positively charged carbon atom on the carbonyl group is attacked, and the resulting intermediate is further dehydrated to form MANG. This process is a classical aldimine condensation reaction. MANG crystallizes in the orthorhombic crystal system with space group P_nn_2 containing four molecules per unit cell. The crystal density is 1.63 g cm^−3^. Its impact sensitivity, detonation velocity and detonation pressure are >7.9 J, 7.1 km s^−1^ and 20.9 GPa, respectively. In addition, we hoped to obtain a more stable five-membered nitrogen heterocyclic compound **59** through the cyclization reaction, but also failed. It illustrates that aldehyde ammonia condensation is more advantageous in this system, and it is difficult to undergo chain reaction and cyclization reaction [68].

**Scheme 19:**
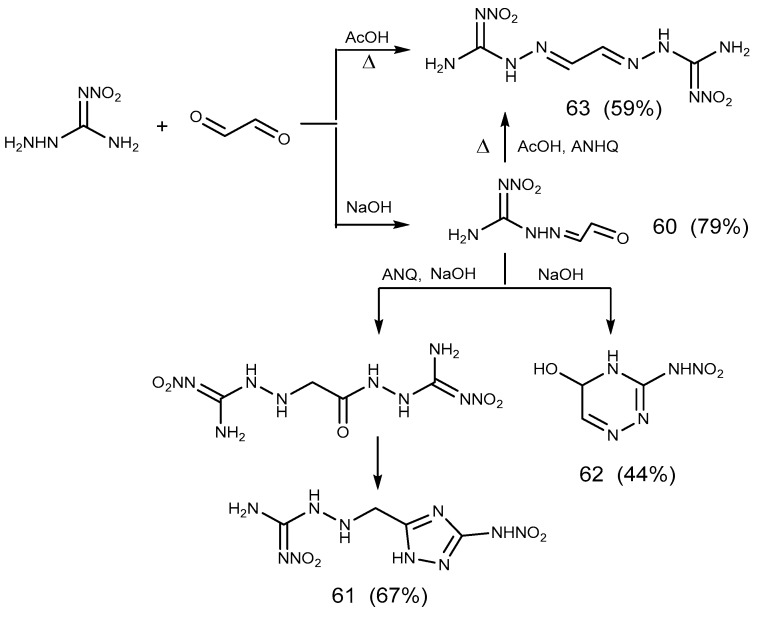
Reaction of ANQ with glyoxal.

**Figure 4:**
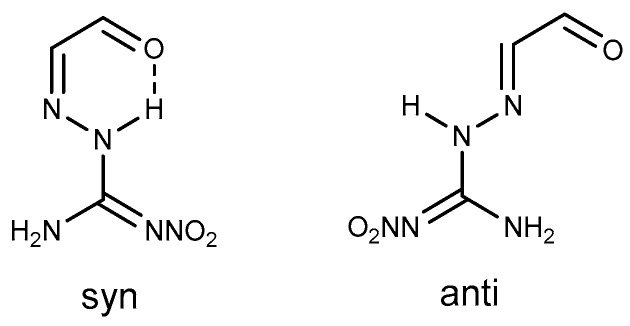
Syn-/anti-isomers of compound **60**.

Treatment of ANQ with glyoxal in sodium hydroxide solution led to a *N*′-nitro-2-(2-oxoethylidene)hydrazinecarboximidamide **(60)** as a mixture of syn and anti-isomers (ratio of 1:1), as seen in Figure 4. When ANQ was 2-fold excess, *N*′-nitro-2-[(5-nitroamino-2*H*-1,2,4-triazol-3-yl) methyl]hydrazinecarboximidamide (**61**) and 3-nitroamino-4,5-dihydro-1,2,4-triazin-5-ol (**62**) were formed. However, under acidic conditions, ANQ reacted with glyoxal to form glyoxal dihydrazone (**63**), which could also be synthesized by hydrazone **60** and ANQ in boiling glacial acetic acid for a long time [69,70], as seen in Scheme 19.

**Scheme 20:**
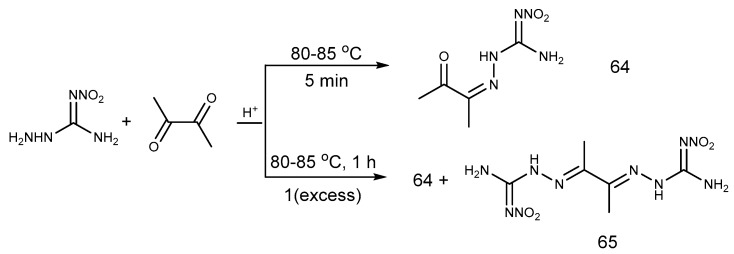
Reaction of ANQ with butane-2,3-dione.

Under the catalysis of acid, a linear monohydrazone (**64**) was prepared by an equimolar ratio of ANQ and butane-2,3-dione. A 2-fold excess of ANQ was used to generate a 5:8 ratio of a mixture of monohydrazone (**64**) and dihydrazone (**65**) [71], as seen in Scheme 20. Similarly, ANQ reacted with acetylacetone to generate acetylacetonenitroguanylosazone [72].

**Scheme 21:**
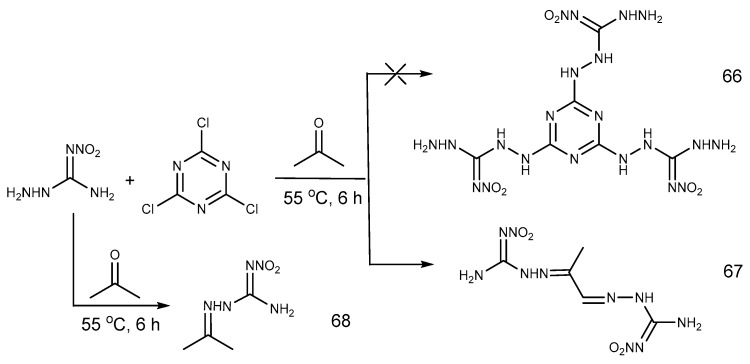
Reaction of ANQ with acetone.

Our group expected that ANQ reacts with trichlorotriazine in acetone to get 2,4,6-tris(1-amino-2-nitroguanidinium)-1,3,5-triazine (**66**), but failed. However, it was surprisingly found that ANQ directly reacted with acetone at 55 °C for 6 h in the presence of trichlorotriazine, which may be catalyst, resulting in the formation of *N*^1^,*N*^2^-bis(2-nitroguanidinium)propane-1,2-diimine (DNPD, **67**). We suspected that trichlorotriazine did not work in the reaction. So we designed the reaction of ANQ only with acetone in the absence of trichlorotriazine and obtained *N*′-nitro-2-(propan-2-ylidene)hydrazine-1-carboximidamide (NPYHC, **68**) successfully, which was a classic reaction of addition condensation reaction of ammonia and ketone. Therefore, trichlorotriazine should play a catalytic role in the reaction, as seen in Scheme 21.

### 2.6. Cyclization Reaction

**Scheme 22:**
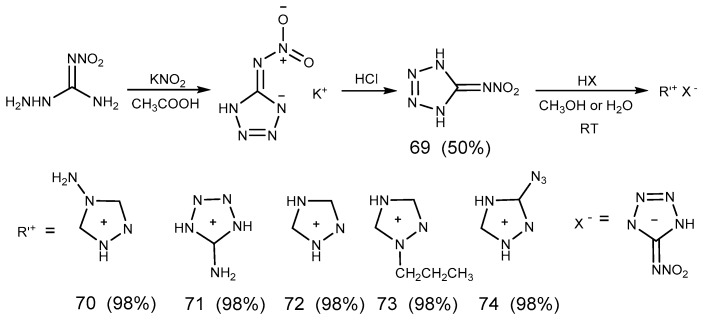
Cyclization reaction of ANQ.

ANQ can undergo many cyclization reactions to synthesize corresponding azoles and azines under different conditions. Compared with traditional carbon-based high-energy compounds such as TNT and TATB, high-energy materials with high nitrogen heterocycles have more advantages, including higher molecular density, fine environmental compatibility and it is easier to achieve good oxygen balance. High nitrogen heterocyclic materials have higher energy and stability due to their high-energy N-N, C-N, N=N bonds and molecular ring strain, so they are more widely used in energetic materials [73]. ANQ was reacted in KNO_2_/AcOH solution firstly, followed by acidifying it with hydrochloric acid to get 5-nitroaminotetrazole (**69**) which melts at 195 °C and explodes at a slightly higher temperature [74,75]. 5-Nitroaminotetrazole is a superior precursor to energetic salts, and the derivatives of **69** have attracted much attention for their positive formation enthalpies and high nitrogen contents [76,77]. Compound **69** can be reduced with benzaldehyde in the presence of Zn to obtain a benzene derivative, or can be reacted with others to obtain corresponding tetrazole ammonium salt [78], diammonium salt [77], methylammonium salt [79], metal salts [80,81,82] (such as lithium salt, sodium salt, rubidium salt, cesium salt) and 1,2,4-triazolium salts [76]. Five different 1,2,4-triazolium salts were synthesized and shown in Scheme 22: 4-amino-1,2,4-triazolium (**70**), 5-amino-tetrazolium (**71**), 1,2,4-triazolium (**72**), 1-propyl-1,2,4-triazolium (**73**) and 3-azido-1,2,4-triazolium (**74**) [76]. Although compound **73** contains energetic anions, its low thermal stability (*T*_m_ = 69 °C) and density (1.48 g cm^−3^) restrict its practical applications. Compound **74** exhibits the highest heat of formation (694.2 kJ mol) and good detonation properties, but the triazolium derivative (**72**) has the highest density in this group compounds [76]. Compared with ordinary tetrazole ions, 5-nitroaminotetrazole is easier to form a corresponding complex with transition metal ions (such as Cu, Ni, Pb, Hg and Ag) [83,84]. These derivatives have potential applications in energetic materials, for instance, using as initiators of explosive materials.

**Scheme 23:**
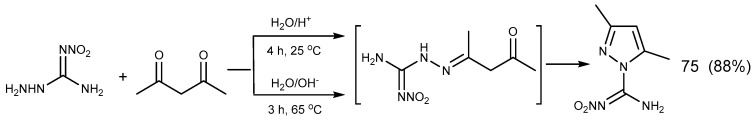
Reaction of ANQ with pentane-2,4-dione.

**Scheme 24:**
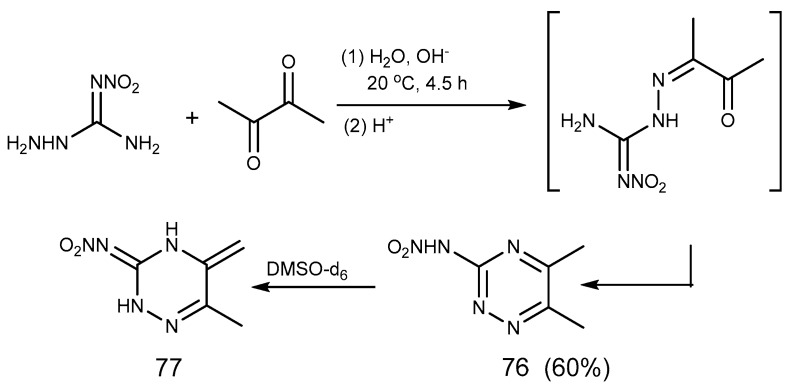
Reaction of ANQ with butane-2,3-dione.

**Scheme 25:**
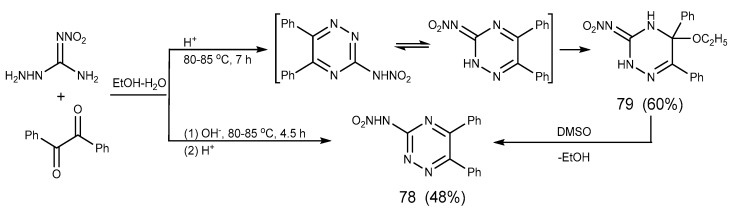
Reaction of ANQ with 1,2-diphenylethanedione.

As long as the reaction temperature and reaction time were properly adjusted, 3,5-dimethyl-*N*-nitro-1*H*-pyrazole-1-carboxamidine **(75)** was formed by reaction of ANQ and pentane-2,4-dione, no matter in acidic or basic conditions [85,86], as seen in Scheme 23. However, ANQ reacted with butane-2,3-dione and 1,2-diphenylethanedione in acid or base conditions leading to different nitroamino products containing 1,2,4-triazine [71]. In water-alkaline solution, ANQ reacted with 2,3-butanedione to form 3-nitroamino-5,6-dimethyl-1,2,4-triazine (**76**) by heterocyclization at room temperature. It existed as a mixture of amine **76** and imine **77** tautomers in a DMSO-*d*_6_ solution, as seen in Scheme 24. Additionally, compounds **64** and **65** were obtained under acidic conditions. In water-alcoholic solution, ANQ reacted with 1,2-diphenylethanedione to obtain 3-nitroamino-5,6-diphenyl-1,2,4-triazine (**78**) at 80 °C for 4.5 h under alkaline condition. When using an acid catalyst, 5-ethoxy-4,5-dihydro-(2*H*)-1,2,4-triazine (**79**) was separated, and the synthesis of **79** could be regarded as the result of the alkoxylation of the initially formed intermediate. Compound **79** was unstable and it could be converted to compound **78** by eliminating ethanol in DMSO-*d*_6_ solution, as seen in Scheme 25 [71].

**Scheme 26:**
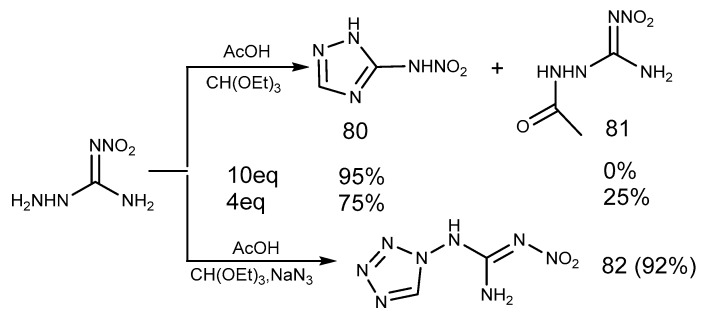
Reaction of ANQ with triethyl orthoformate.

**Scheme 27:**
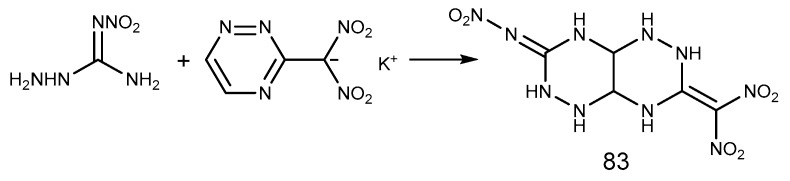
Reaction of ANQ with the derivative of FOX-7.

ANQ condensed with triethyl orthoformate to form 3-nitramino-1,2,4-triazole (**80**). When the dosage of orthoformate was reduced, the hydrazino group of ANQ was acetylated to form *N*-acetyl-3-amino-1-nitroguandine (**81**). However, compound **81** was unstable and converted to another product easily. In the presence of sodium azide, 1-nitroguanidyltetrazole (**82**) was prepared by ANQ and triethyl orthoformate in glacial acetic acid medium [87], as seen in Scheme 26. Furthermore, the condensation reaction of ANQ with heterocyclic 3-(dinitromethyl)-1,2,4-triazine potassium salt could result in the corresponding compound, 3-nitrimino-7-dinitromethylene-octahydro-(1,2,4)triazino-(6,5-e) (1,2,4) triazine (**83**), as seen in Scheme 27. Its detonation properties (*P* = 29.4 GPa, *D* = 8.492 km s^−1^) compare favorably with those of ANQ, so it has potential application value in energetic materials [88].

### 2.7. Azide Reaction

**Scheme 28:**
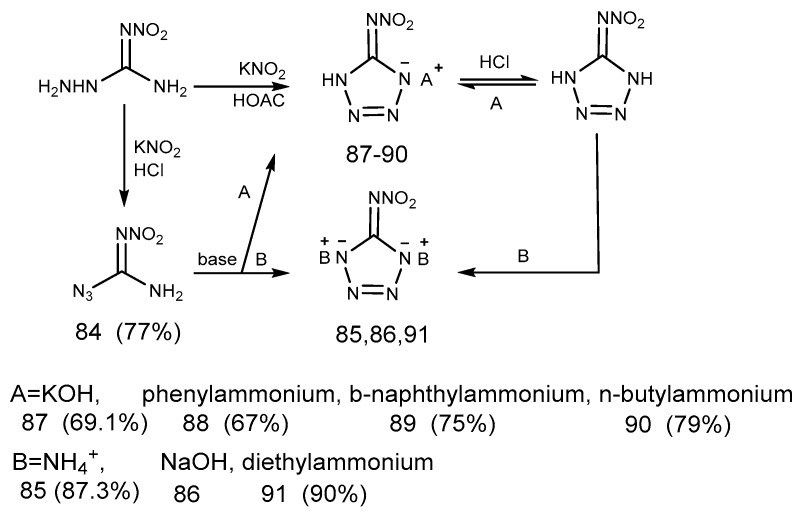
Azide reaction of ANQ.

ANQ reacted in a strong acid solution of HCl/KNO_2_ to obtain azide nitroguanidine (**84**) at 60 °C with the yield of 77% [75]. It was found that ANQ could react with nitrous acid in weak or strong acid solution to obtain azide nitroguanidine, and the yield in strong acid solution was higher. Compound **84** has a high nitrogen content (64.62%) and presents a potential application value. It can be reduced to nitroguanidine by hydrogen sulfide, as a characteristic reaction of azide. Compound **84** was cyclized with inorganic base or organic base to obtain the corresponding alkyl or aryl ammonium salts of 5-nitroaminotetrazole (**85**–**91**), which could also be obtained directly by alkalizing 5-nitroaminotetrazole, as seen in Scheme 28 [74].

### 2.8. Detonation Properties

A series of derivatives of ANQ were summarized systemically and some compounds, as potential energetic materials, display good energetic properties, as shown in Table 2. We use the classification in “UN Recommendations on the Transport of Dangerous Goods” to evaluate their safety [89]. Compound **27** is the most sensitive compound with values of 1 J (IS) and 20 N (FS), which is classified as very sensitive. The other compounds except the compound **35** (>40 J) and **36** (>60 J) are sensitive. However, compounds **22**, **33**, **34**, **38**, **39**, **41**, **42**, **58** and **83** exhibit impact sensitivities between 7–10 J, and they are comparable to or less sensitive than RDX. Compounds **50**, **51**, **67** and **68** are considerably less sensitivity than RDX, but similar to ANQ. Regarding the friction sensitivity some compounds follow the trends observed for the impact sensitivities, some do not. For example, compound **83** (>360 N) can be classified as an insensitive material to friction, and compounds **22**, **26**, **41**, **50** and **51** have similar friction sensitivity to ANQ and RDX, but all of them have higher friction sensitivity than TNT. For compounds **23**–**25**, even if they are less sensitive, the decomposition temperatures of them are only 80 °C, so they are of little value for energetic applications. The detonation pressures (*P*) of these derivatives distribute in the range of 19.06–48.5 GPa, and their detonation velocities (*V*_det_) are found in the range from 6.860 to 10.358 km s^−1^, most of which are between 8.230–9.551 km s^−1^. Compound **69** shows the highest detonation velocity (10.358 km s^−1^) and the highest detonation pressure (48.5 GPa), and the detonation velocities of its derivatives (**70**–**74**) are comparable to that of RDX. The detonation performances of compounds **22**, **33**, **38**–**42** and **69** exceed that of RDX except compound **40**. Compound **35**, **50**, **58** and **68** reach the energy level of RDX, and all of the other compounds show comparable or superior detonation velocities to that of ANQ. Compound **30** (*P* = 26.6 GPa and *V*_det_ = 8.050 km s^−1^), **35** (*P* = 23.4 GPa and *V*_det_ = 7.500 km s^−1^) and **58** (*P* = 20.9 GPa and *V*_det_ = 7.100 km s^−1^) possess relatively poor detonation performances for their lower densities of 1.59–1.63 g cm^−3^ compared to the other compounds (1.70–2.06 g cm^−3^), supporting the view that high density contributes markedly to the detonation performances. In summary, the compounds, **22**, **33**, **38**–**42** and **69** exhibit good detonation properties, and can be regarded as potential candidates in the application of high-energy-density materials.

## 3. Conclusions

Since the first report, ANQ has been regarded as an important raw material in the fields of pesticides, medicines and energetic materials. As a high-energy insensitive material, ANQ is likely to replace traditional RDX in application of solid propellants.

Though the molecular structure is very simple, its reactivity is abundant. Seven kinds of reactions about ANQ are systematically summarized, including reduction reaction, acylation reaction, salification reaction, coordination reaction, aldimine condensation reaction, cyclization reaction and azide reaction. Many excellent derivatives have been synthesized by these reactions. Some high-energy nitrogen-rich derivatives, such as ANGN, 1-amino-2-nitroguanidinium 5,5′-bis(tetrazole-2-oxide) salt and 5-nitroaminotetrazole, exhibit a good application prospect in the field of energetic materials.

According to the summary of the reactivity for ANQ, we can see that the adjacent amino and hydrazino group is a high activity group and the key factor for synthesizing these derivatives, especially to the heterocyclic derivatives. Therefore, in the design of nitrogen-rich or heterocyclic compounds, the introduction of the adjacent amino and hydrazino group is an effective way. We believe that many new nitrogen-rich materials can be synthesized according this method. The research of the reactivity of ANQ contributes significantly to expanding the understanding of the chemistry of guanidine compounds.

## Figures and Tables

**Table 1:** General and detonation performance comparison of ANQ, NQ, RDX, TNT, and FOX-7.

	ANQ	NQ	RDX	TNT	FOX-7
*M*^a^ (g mol^−1^)	119.08	104.07	222.12	227.13	148.08
*ρ*^b^ (g cm^−3^)	1.767	1.759	1.80	1.663	1.885
∆_f_*H* ^c^ (kJ mol^−1^)	161.7	−86	70	−59.4	−32.0
*T*_dec_^d^ (°C)	184	254	205	295	237.2
*P*^e^ (GPa)	30.7	29.0	34.9	19.1	28.4
*V*_det_^f^ (m s^−1^)	8250	8344	8795	6928	8325
IS ^g^ (J)	20	>50	7.5	39.4 ^n^	24.7
FS ^h^ (N)	144	>355	120	>353 ^o^	>350
ESD ^i^ (J)	0.15	-	0.2	0.25	0.45
N ^j^ (%)	58.8	53.83	37.84	18.5	43.24
OB ^k^ (%)	−33.59	−30.75	−21.6	−74.0	−21.61
Reference	[18] ^l^,[21,22]	[23] ^m^	[24],[25] ^l^,[26]	[26,27,28,29]	[30,31,32,33,34]

^a^ Molecular mass. ^b^ Crystal density. ^c^ Molar enthalpy of the formation of compound. ^d^ Decomposition temperature. ^e^ Detonation pressure. ^f^ Detonation velocity. ^g^ Impact sensitivity. ^h^ Friction sensitivity. ^i^ Sensitivity to the electrostatic discharge. ^j^ Nitrogen content. ^k^ Oxygen balance. ^l^ IS: STANAG 4489 modified instructions, BAM Drop Hammer; FS: STANAG 4487 modified instructions, BAM friction tester; ESD: OZM, the Electric Spark Tester ESD 2010 EN. ^m^ IS: the BAM test (UN Test Series 3(a)(ii), with the 30 kg Fall Hammer-test (UN Test series 3(a)(iv). ^n^ IS: h_50_ of 160 cm, 2.5 kg Hammer. ^o^ FS: BAM friction tester.

**Table 2:** Physical and energetic properties of some derivatives of ANQ.

	IS ^a^ (J)	FS ^b^ (N)	ESD ^c^ (J)	*T*_dec_^d^ (°C)	∆_f_*H* ^e^ (kJ mol^−1^)	*M*^f^ (g mol^−1^)	N ^g^ (%)	*ρ*^h^ (g cm^−3^)	*T*_det_^i^ (K)	*P*^j^ (GPa)	*V*_det_^k^ (m s^−1^)	Ref.
22	10 ^l^	120	0.50	130	563.4	182.10	46.2	1.905	4196	42.7	9551	[18] ^l^
23	25	288	0.15	80	-	173.56	39.82	1.731	-	-	-	[48] ^l^
24	25	288	0.20	80	-	218.03	31.76	2.024	-	-	-	[48] ^l^
25	25	288	0.20	86	-	265.02	26.46	2.339	-	-	-	[48] ^l^
26	6	120	0.30	144	-	336.29	41.35	1.968	-	-	-	[48] ^l^
27	1	20	0.15	130	599.4	219.54	31.90	1.980	-	-	-	[18] ^l^
28	-	-	-	143.0	264.7	-	43.58	1.70	-	30.2	8398	[49]
29	-	-	-	121.6	487.2	-	59.82	1.64	-	29.1	8334	[49]
30	-	-	-	175.5	551.3	-	56.49	1.59	-	26.6	8050	[50]
31	-	-	-	217.1	353.7	-	50.44	-	-	29.8	8420	[51]
32	-	-	-	-	467.0	590.42	-	-	-	31.8	8642	[52]
33	10	40	0.10	108	511.4	244.1	45.9	1.850	3949	37.7	9175	[18] ^l^
34	12	228	0.20	118	40.9	313.09	44.7	1.705	3612	31.1	8656	[18] ^l^
35	>40	-	-	-	-	347.2	32.09	1.63	-	23.4	7500	[22] ^m^
36	>60	-	-	202.1	337.5	248.06	42.41	1.75	-	30.7	8392	[54] ^n^
37	5	96	0.40	136	544.3	285.2	54.4	1.729	3607	31.7	8753	[18] ^l^
38	10	48	1.5	163	1043.8	408.26	61.76	1.832	4129	38.2	9350	[55] ^l^
39	7	54	-	109	986.6	-	66.2	1.79	-	36.1	9432	[56] ^o^
40	-	-	-	171	557.1	-	-	1.72	-	33.7	9171	[57]
41	7	120	0.07	149	593.1	584.29	43.15	1.85	4343	36.6	8872	[58] ^l^
42	8	80	-	130	450.0	-	-	1.88	-	39.5	9237	[59] ^o^
50	19	128	0.268	218	421.6	-	42.8	1.65	-	24.8	7670	[63] ^o,p^
51	24	168	0.305	204	306.2	-	32.8	1.79	-	28.1	7965	[63] ^o,p^
58	>7.9	-	-	170.9	-	-	-	1.63	-	20.9	7100	[68] ^q^
67	>21.56	-	-	-	676.95	-	-	1.690	-	28.43	8390	
68	19.6	-	-	-	−470.9	-	-	1.555	-	19.06	6860	
69	-	-	-	-	252.3	-	64.12	2.06	-	48.5	10,358	[76]
70	-	-	-	184	440.6	-	65.4	1.72	-	27.0	8506	[76]
71	-	-	-	165	487.5	-	71.6	1.63	-	25.5	8276	[76]
72	-	-	-	177	328.0	-	63.3	1.74	-	26.0	8334	[76]
74	-	-	-	135	694.2	-	70.0	1.68	-	25.9	8230	[76]
83	8	360	-	123.5	304.2	-	-	1.73	-	29.4	8492	[88] ^o^
ANQ	20	144	0.15	184	161.7	119.08	58.8	1.767	-	30.7	8250	[18] ^l^, [21,22]
RDX	7.5	120	0.2	205	70	222.12	37.84	1.80	-	34.9	8795	[24], [25] ^l^, [26]
TNT	39.4 ^r^	>353 ^o^	0.25	295	−59.4	227.13	18.5	1.663	-	19.1	6928	[26,27,28,29]

^a^ Impact sensitivity. ^b^ Friction sensitivity. ^c^ Sensitivity to the electrostatic discharge. ^d^ Decomposition temperature. ^e^ Molar enthalpy of the formation of compound. ^f^ Molecular mass. ^g^ Nitrogen content. ^h^ Crystal density. ^i^ Explosion temperature. ^j^ Detonation pressure. ^k^ Detonation velocity. ^l^ IS: STANAG 4489 modified instructions, BAM Drop Hammer; FS: STANAG 4487 modified instructions, BAM friction tester; ESD: OZM, the Electric Spark Tester ESD 2010 EN. ^m^ IS: ZBL-B impact sensitivity instrument, 2.0 kg Drop Hammer. ^n^ IS: 5.0 kg Drop Hammer. ^o^ IS: standard BAM Fall Hammer; FS: BAM friction tester. ^p^ ESD: Electric Spark Tester ESD JGY-50 III. ^q^ IS: 5.0 kg Drop Hammer. ^r^ IS: h50 of 160 cm, 2.5 kg Hammer.
